# Assessment of the feasibility and safety of totally implantable venous access port to collect blood samples in pediatric patients with acute lymphoblastic leukemia

**DOI:** 10.12669/pjms.41.6.11978

**Published:** 2025-06

**Authors:** Yanqing Wang, Meiying Gao, Guannan Bai

**Affiliations:** 1 Yanqing Wang Department of Hematology-Oncology, Children’s Hospital, Zhejiang University School of Medicine, National Children’s Regional Medical Center, National Clinical Research Center for Child Health, Hangzhou, Zhejiang Province, P.R. China; 2 Meiying Gao Department of Child Health Care, Children’s Hospital, Zhejiang University School of Medicine, National Children’s Regional Medical Center, National Clinical Research Center for Child Health, Hangzhou, Zhejiang Province, P.R. China; 3 Guannan Bai Department of Child Health Care, Children’s Hospital, Zhejiang University School of Medicine, Children’s Hospital, Zhejiang University School of Medicine, National Children’s Regional Medical Center, National Clinical Research Center for Child Health, Hangzhou, Zhejiang Province, P.R. China

**Keywords:** Child, Methotrexate, Precursor Cell Lymphoblastic Leukemia-Lymphoma, Vascular Access Devices

## Abstract

**Objective:**

To assess the feasibility and safety of utilizing a totally implantable venous access port (TIVAP) to collect venous blood sample as an alternative to peripheral venipuncture in pediatric patients with acute lymphoblastic leukemia (ALL).

**Method:**

The study implemented a self-contemporaneous control design. Fifty pediatric patients diagnosed with ALL were recruited from a tertiary children’s hospital between January to November 2024 in Hangzhou, China. They underwent high-dose methotrexate (HD-MTX) chemotherapy. For each participant, both TIVAP and peripheral venipuncture were used to collect blood samples. The MTX concentration, levels of blood indicators, coagulation function and adverse events were measured and compared between TIVAP and peripheral venipuncture groups.

**Results:**

The mean age was 6.1 (standard deviation: 2.9) years. No statistically significant difference in MTX plasma concentration was observed between TIVAP and peripheral venipuncture group at 24, 48, and 72 hours in all patients and subgroups of low, intermediate and high risk (*p* values > 0.05). In addition, we did not observe statistically significant differences (*p* values > 0.05) in levels of blood indicators (i.e., serum creatinine, alanine aminotransferase, white blood count, hemoglobin, platelet, pH, serum potassium, sodium, chloride, and lactate) and coagulation function profiles (i.e., prothrombin time, activated partial thromboplastin time, thrombin time, fibrinogen and plasma D-dimer) between TIVAP and peripheral venipuncture group. No adverse events, such as hemolysis or coagulation issues, were observed in the TIVAP group.

**Conclusions:**

TIVAP had a comparable feasibility and safety with peripheral venipuncture, and may be used as an alternative to collect blood sample in pediatric patients with ALL.

## INTRODUCTION

Acute lymphocytic leukemia (ALL) is among the most common pediatric cancers. The current cure rate was nearing 90%^1^, but some children may have the challenge of relapse.^2^ High-dose methotrexate (HD-MTX) is pivotal in preventing extramedullary leukemia in pediatric patients with ALL.^3^ Despite its substantial anti-tumor efficacy, HD-MTX is associated with various adverse effects, including acute kidney injury, severe mucositis, bone marrow suppression, gastrointestinal disturbances, hepatotoxicity, and leukoencephalopathy.^4^ To maximize the therapeutic effects of HD-MTX, it is crucial to regularly monitor drug concentrations via venous blood sampling every 24 hours. This practice facilitates the appropriate adjustment of calcium folinate dosages to maintain effective therapeutic levels and enhance efficacy until the methotrexate plasma concentration decreases to a safe threshold (< 0.2 μmol/L).^5^-^7^ Simultaneously, monitoring of blood counts, liver and kidney function, electrolytes, and coagulation profiles is necessary to evaluate the patient’s condition. Currently, peripheral venipuncture remains the standard method for blood collection in clinical practice.

However, frequent venous punctures not only cause pain or discomforts for pediatric patients with ALL, but also result in complications such as needle or blood phobia, subcutaneous hematomas, local bleeding, accidental arterial puncture, failed insertions, and hemolysis.^8^ The totally implantable venous access port (TIVAP), a novel central vascular access device (CVAD), could reduce the above-mentioned discomforts and complications, and is increasingly utilized for chemotherapy, blood transfusions, intravenous nutrition, antibiotic administration, and other infusions, as well as for blood sample collection.^9^ Recent guidelines underscore the dual functionality of TIVAPs in the administration of chemotherapy and the blood sampling, with particular emphasis on pediatric populations requiring long-term treatment durations.^9^ The multidisciplinary expert consensus on implantation and management of venous infusion port addresses the benefits of TIVAPs in minimizing procedural trauma relative to peripheral venipuncture.^10^ Notably, in the context of HD-MTX administration, it requires precise pharmacokinetic monitoring with frequent blood sampling every 24 hours due to the narrow therapeutic window. Frequent blood samplings may lead to procedural trauma in pediatric patients vulnerable to HD-MTX toxicity, increasing the risk of phlebitis, hemolysis, and psychological distress, which can influence treatment adherence and effectiveness. So, TIVAP may be used as essential approach to overcome the above-mentioned barriers in the clinical setting. Despite its rising application, there is a lack of studies that specifically address blood sampling from the lumen of TIVAP used for drug monitoring during infusions.

This study evaluated the feasibility and safety of TIVAP for HD-MTX concentration monitoring in pediatric ALL patients, aiming to establish evidence-based practices for blood sampling via CVADs while minimizing procedural trauma.

## METHOD

This study was conducted within the Department of Hematology-Oncology at the Children’s Hospital, Zhejiang University School of Medicine.

### Inclusion criteria:

- A confirmed diagnosis of ALL.
- Administration of HD-MTX chemotherapy.
- No symptoms of blood infection.
- The diagnosis protocol and the risk classification criteria were presented in Supplementary Table-SI.

**Supplementary Table-I:** ECPHOG-B-ALL-2024 Newly Diagnosed ALL Classification Criteria.

Low-risk group: All of the following criteria must be met:	Intermediate-risk group: one or more of the following criteria must be met:	High-risk group: one or more of the following criteria must be met:
1. Age ≥ 1 year and <10 years 2. White blood cell count (WBC) <50×10^9^/L 3. Absence of CNS2, CNSL (CNS3), and/or testicular leukemia (TL) 4. No adverse cytogenetic and molecular biological features characteristic of intermediate and high-risk groups. 5. Bone marrow at M1 stage (lymphoblastic +prolymphocytic <5%) on day 15 of induction chemotherapy, with M1 status also maintained at the end of induction therapy 6. No signs of leukemic infiltration, and complete resolution of mediastinal tumor foci at the end of induction therapy, as assessed by clinical and imaging evaluation 7. MRD criteria: Multicolor flow cytometry (MFC) criteria: MRD < 1 × 10^-3^ on day 15, and MRD < 1 × 10^-4^ on day 29 at the conclusion of induction therapy. For children positive for ETV6/RUNX1, those with a day 29 MRD < 1 × 10^-4^, despite a day 15 MRD between 1 × 10^-3^ and 1 × 10^-2^, remain classified as low-risk. 8. NGS-MRD criteria: MRD < 1 × 10^-4^ on day 29 at the end of induction therapy and MRD < 1 × 10^-6^ at the end of consolidation therapy (week 12).	1. Age ≥ 10 years 2. Maximum WBC ≥ 50×10^9^/L at initial diagnosis 3. Presence of CNS2, CNSL (CNS3), and/or testicular leukemia (TL) 4. E2A-PBX1/t (1; 19) 5. B-cell type on day 15 of induction, required to achieve bone marrow M1 status. 6. T-ALL 7. iAMP21 8. Ph+ALL 9. Ph-like ALL 10. At the end of induction therapy, mediastinal tumor foci that have not completely resolved, but have reduced to < 1/3 of their initial volume 11. MRD criteria: MFC MRD on day 15 of induction: 1 × 10^-3^ ≤ MRD < 1 × 10^-2^, or at the end of induction therapy (day 29): 1 × 10^-4^ ≤ MRD < 1 × 10^-2^ (3) NGS-MRD at the end of induction (day 29): MRD ≥ 1 × 10^-4^, or at the end of consolidation therapy (week 12): MRD ≥ 1 × 10^-6^.	1. B-cell type on day 15 of induction: bone marrow M2 status 2. Incomplete remission (M2 or M3, with lymphoblastic +prolymphocytic forms ≥ 5%) at the end of induction therapy (days 29-33) 3. Positive for MLL gene rearrangement 4. Hypodiploidy (≤ 44) or a DI index < 0.8 5. Homozygous, large fragment deletion of IKZF1 6. MEF2D rearrangement 7. TCF3-HLF/t(17;19)(q22;p13) 8. Mediastinal tumor foci not reduced to < 1/3 of initial volume at the end of induction therapy (days 29-33) 9. MRD criteria: (1) MRD ≥ 1 × 10^-2^ on day 15 of induction or MRD ≥ 1 × 10 ^-2^ at the end of induction therapy (day 29) (2) NGS-MRD ≥ 1 × 10^-4^ at the end of consolidation therapy (week 12). (3) NGS-MRD ≥ 1 × 10^-6^ after 12 weeks of blinatumomab treatment.

### Exclusion criteria:

- Poor family compliance.
- Presence of infection symptoms.
- Not willing to participate in the study.
- Unable to complete treatment due to the disease.
- A discrepancy exceeding one minute in blood sample collection times between TIVAP and peripheral venipuncture.

Informed written consent was obtained from the guardians of all participating children before conducting the study. In total, 50 patients diagnosed with ALL were recruited from January to November 2024. The mean age was 6.1 years and the standard deviation was 2.9 years. Twenty-eight (56.0%) were boys and 22 (44.0%) were girls.

### Ethical Approval:

The study was conducted in accordance with the Declaration of Helsinki, and was approved by the Ethics Committee of Children’s Hospital, Zhejiang University School of Medicine (approval number: 2023-IRB-0254-P-01; approval date: October 27, 2023).

The study implemented a self-contemporaneous control design. Each participant served as their own control by undergoing both TIVAP and peripheral venipuncture sampling during the same HD-MTX treatment cycle. This approach minimizes inter-individual variability (e.g., age, pharmacokinetic differences) and enhances statistical power by directly comparing paired samples from the same patient under standardized conditions. During HD-MTX chemotherapy, methotrexate was administered via continuous infusion over a 24-hour period, with blood samples collected at 24, 48, and 72 hours after the methotrexate infusion.^11^ In a single patient, blood samples were collected by a well-trained nurse using peripheral venipuncture and TIVAP respectively, with the two sampling procedures performed within one minute of each other. The blood sample collected by TIVAP is called the experimental sample, and the blood sample collected by the standard approach is called the control sample.

The experimental samples were collected via the push-pull technique through a TIVAP infused with HD-MTX and electrolyte solution. The protocol included discontinuing fluid infusion, removing the infusion set, disinfecting the positive pressure connector with an alcohol swab, and aspirating three times the total volume of the port system and its attachments (0.97mL: port chamber 0.15 mL, untrimmed catheter 0.4mL, non-coring needle 0.2mL, positive pressure connector 0.22 mL) using a 10 mL sterile syringe. The aspirated blood was slowly reinjected, and this process was repeated for four cycles. Both aspiration and reinjection were performed at a slow and steady rate to avoid hemolysis caused by excessive force on the plunger.

Subsequently, a new 10mL sterile syringe was used to collect the required blood specimens, which were gently transferred along the tube wall into a vacuum blood collection tube after adequate withdrawal. The positive pressure connector was disinfected again with an alcohol swab, followed by pulsatile flushing of the catheter with 20mL normal saline. Finally, the infusion set was reconnected to resume fluid administration. The control samples were collected following the standard blood collection protocol,^8^ with prolonged compression duration appropriately applied for children with coagulation dysfunction.^12^ Experimental and control samples were then simultaneously transported to the Clinical Laboratory Center in the hospital for analysis using the same instrument. The control group results were retrieved from medical records, whereas the laboratory results of the experimental samples were directly obtained from the Clinical Laboratory Center.

### Adverse events:

Based on the four-week maintenance protocol for TIVAP during inter-treatment intervals, pediatric patients were prospectively monitored for TIVAP-associated complications (e.g., port dysfunction, catheter-related bloodstream infection) for 28 days following paired blood sample collection. Diagnostic classification of complications was based on the Expert Consensus on Implantable Drug Delivery Systems.^13^

### Statistical analysis:

Statistical analyses were conducted using SPSS version 27.0. Tests for normality were applied to the data; variables with a normal distribution were reported as “mean ± standard deviation,” and comparisons between groups were made using the independent samples *t*-test. For data not following a normal distribution, results were expressed as “median (interquartile range),” indicating the 25th and 75th percentiles, with inter-group comparisons conducted using the Mann-Whitney U test. A *p*-value of less than 0.05 indicates statistical significance.

## RESULTS

For 50 participants, the mean age was 6.1 years and the standard deviation was 2.9 years. Twenty-eight (54.0%) were boys and twenty-two (46.0%) were girls. Twenty nine out of 50 (58.0%) participants lived in the urban areas. 70.0% of parents’ educational level was high school or above. The proportions of children with low, intermediate, and high risk of ALL are 40.0%, 40.0% and 20.0%, respectively. The median of disease duration was 159.0 (interquartile range: 75.5, 238.0) days.

Table-I presents the differences in MTX plasma concentrations at 24, 48 and 72 hours between TIVAP and peripheral venipuncture group in all patients and in subgroups of children with low, intermediate and high risk. No statistically significant difference in MTX plasma concentrations was observed between these two groups at 24, 48, and 72 hours in all patients and subgroups (*p* values > 0.05).

**Table-I:** Comparison of MTX concentrations (μmol/L) at 24, 48 and 72 hours between TIVAP and peripheral venipuncture groups.

	24 hours			48 hours			72 hours		
	Venipuncture	TIVAP	p	Venipuncture	TIVAP	p	Venipuncture	TIVAP	p
All (N = 50)	67.11 (52.48, 82.57)	64.38 (50.15, 80.34)	0.53	0.69 (0.51, 0.89)	0.68 (0.50, 0.88)	0.92	0.15 (0.12, 0.22)	0.15 (0.11, 0.21)	0.76
Low-risk (n = 20)	52.29 (49.00, 63.30)	49.89 (47.69, 61.53)	0.30	0.57 (0.48, 0.76)	0.56 (0.48, 0.75)	0.91	0.13 (0.10, 0.20)	0.12 (0.10, 0.19)	0.80
Intermediate-risk (n = 20)	67.98 (62.36, 87.63)	66.85 (59.89, 87.30)	0.71	0.80 (0.60, 0.99)	0.80 (0.61, 0.97)	0.89	0.17 (0.14, 0.24)	0.16 (0.14, 0.23)	0.65
High-risk (n = 10)	88.18 (78.16, 94.64)	87.61 (76.29, 93.45)	0.88	0.85 (0.42, 1.03)	0.85 (0.41, 1.04)	1.00	0.16 (0.11, 0.28)	0.16 (0.11, 0.28)	0.91

Table-II presents the blood indicators including serum creatinine, alanine aminotransferase, white blood count, hemoglobin, platelet, pH, serum potassium, sodium, chloride, and lactate levels between TIVAP and peripheral venipuncture groups. There is no statistically significant difference in the levels of the above blood indicators between these two groups (*p*-values > 0.05).

**Table-II:** Comparison of blood indicators at 48 hours between TIVAP and peripheral venipuncture groups.

Variables	Venipuncture	TIVAP	p
SCR (μmol/L)	32.86 ± 7.52	32.64 ± 7.96	0.89
ALT (U/L)	31.00 (24.00, 42.00)	31.50 (23.75, 41.00)	0.94
WBC (×10^9^/L)	3.61 (2.98, 4.58)	3.54 (2.95, 4.55)	0.81
Hb (g/L)	90.00 (81.75, 98.00)	87.50 (79.75, 96.00)	0.45
PLT (×10^9^/L)	233.50 (166.25, 287.50)	230.50 (168.75, 312.00)	0.86
pH	7.38 (7.36, 7.40)	7.38 (7.36, 7.40)	0.96
K+ (mmol/L)	3.90 (3.70, 4.20)	4.10 (3.70, 4.20)	0.39
Na+ (mmol/L)	139.12 ± 2.98	139.94 ± 2.69	0.15
Cl－ (mmol/L)	99.50 (98.00, 104.25)	103.50 (99.00, 106.00)	0.07
Lactic acid (mmol/L)	1.90 (1.60, 2.42)	1.95 (1.60, 2.42)	0.98

P values were calculated by the independent samples t-test or the Mann-Whitney U test.

***Abbreviations:*** SCR, serum creatinine; ALT, alanine aminotransferase; WBC, white blood count; Hb: Hemoglobin; PLT, platelet.

Table-III presents the coagulation function profiles between TIVAP and peripheral venipuncture groups. There is no statistically significant difference regarding prothrombin time (PT), activated partial thromboplastin time (APTT), thrombin time (TT), fibrinogen (FIB) and plasma D-dimer between these two groups (*p*-values > 0.05).

**Table-III:** Comparison of coagulation function profiles at 48 hours between TIVAP and peripheral venipuncture groups.

Variables	Venipuncture	TIVAP	Statistic	p
PT (s)	10.67 ± 0.66	10.75 ± 0.64	t = -0.57	0.57
APTT (s)	26.35 (25.38, 32.20)	26.45 (25.45, 32.15)	Z = -0.20	0.84
TT (s)	18.10 (17.40, 19.33)	18.10 (17.48, 19.40)	Z = -0.17	0.87
FIB (g/L)	2.38 (1.96, 2.76)	2.37 (1.98, 2.77)	Z = -0.13	0.89
D-dime (mg/L)	0.37 (0.23, 0.58)	0.38 (0.24, 0.58)	Z = -0.23	0.82

P values were calculated by the independent samples t-test or the Mann-Whitney U test.

***Abbreviations:*** PT, prothrombin time; APTT, activated partial thromboplastin time; TT, thrombin time; FIB, fibrinogen.

At the 28 days post-collection follow-up, there was no TIVAP-associated complications (e.g., catheter obstruction, catheter-related bloodstream infection) and no preanalytical specimen integrity issues (hemolysis or coagulation artifacts) in the experimental group. In contrast, two blood samples in the control group demonstrated coagulation profile abnormalities across complete blood count, electrolyte, and coagulation function panels, and we repeated sampling to ensure analytical validity.

## DISCUSSION

The present study has evaluated the clinical feasibility and safety profile of TIVAP-mediated venous blood sampling as a non-inferior alternative to peripheral venipuncture in pediatric ALL patients receiving infusion therapy. Longitudinal pharmacokinetic analysis demonstrated no statistically significant intergroup differences in MTX plasma concentrations, and there was no statistically significant difference in hematological parameters, electrolyte profiles, and coagulation function between TIVAP and peripheral venipuncture group.

The adverse effects of HD-MTX significantly compromise therapeutic outcomes and impair quality of life in pediatric patients, so it is necessary to frequently monitor the serum MTX concentrations to ensure treatment safety. Current infusion practice standards stipulate that therapeutic drug monitoring (TDM) specimens should ideally be obtained from a dedicated lumen not utilized for administration of the monitored medication.^9^ Consequently, in clinical nursing protocols, while HD-MTX is administered via the TIVAP, MTX plasma concentration monitoring routinely requires peripheral venipuncture. However, pediatric venous accessibility limitations compounded by repeated venipuncture attempts frequently result in procedural failures. This study demonstrates that blood sample collection through TIVAPs previously infused with HD-MTX and electrolyte solutions effectively circumvents these clinical challenges.

The bolus-aspiration technique represents a clinically validated method for blood sample collection through CVADs. Prior studies have established that aspirating four-six mL of blood into a syringe followed by four cycles of controlled push-pull maneuvers with subsequent reinjection optimizes specimen reliability.^14^-^16^ In this study, we implemented a modified protocol: three times the total internal volume (0.97 mL) of the port system (approximately 3 mL) was aspirated per cycle, completing four complete push-pull sequences. This resulted in a total blood displacement volume of 12 mL, exceeding the 10 mL threshold recommended by infusion standards for effectively clearing fibrin deposits, drug precipitates, and residual MTX from TIVAP lumens. Notably, we demonstrated superior safety with our protocol by reducing hemolysis risks compared to conventional methods requiring larger displacement volumes in the above-mentioned prior studies.^14^-^16^

The present study demonstrated that MTX plasma concentrations at 24-, 48-, and 72-hour intervals showed comparable values between the control and experimental groups in pediatric patients with low-, intermediate-, and high-risk ALL, with no statistically significant differences observed (*p* values > 0.05). In addition, comprehensive clinical indicators exhibited remarkable consistency between the two groups. These findings collectively supported the clinical feasibility of utilizing TIVAP for blood sample collection in HD-MTX chemotherapy.

Our study further validated the clinical safety profile of using TIVAP that was previously administered HD-MTX and electrolyte solutions to collect blood samples for MTX plasma concentration monitoring and other clinical tests. We did not observe TIVAP-related complications or hemolysis in the experimental group. It is important to thoroughly flush the TIVAP chamber and its accessories, because catheter occlusion is predominantly caused by blood reflux and subsequent clot formation within the catheter lumen^10^, while catheter-related bloodstream infections are associated with endogenous microorganisms from colonized catheter surfaces.^17^ Notably, a recent study comparing TIVAPs and peripherally inserted central catheters (PICCs) in pediatric patients with Hodgkin lymphoma demonstrated significantly lower rates of severe complications with TIVAPs, including catheter-related central venous thrombosis (CVT) (7% vs. 12%)^18^. Our results, combined with comparative data from van der Bosch^18^, support using TIVAPs over PICCs in pediatric ALL patients needing frequent blood samplings. The reduced CVT risk with TIVAP improves safety and minimizes treatment disruptions, caused by catheter failure that is crucial for maintaining HD-MTX pharmacokinetic accuracy and reducing procedural trauma. The results of this study support the updated clinical guidelines recommending the use of TIVAPs in pediatric patients with thrombotic risk factors.

In this study, we implemented a pulsed irrigation protocol using 20 mL of normal saline to effectively remove residual blood from the TIVAP system. Subsequently, continuous infusion was maintained via an infusion set. During the four-week observation period, no TIVAP-related complications such as catheter occlusion or infections occurred. None of the experimental blood sample exhibited adverse events, including hemolysis or coagulation. These findings support the safety of TIVAP to collect blood samples.

To our best knowledge, it was one of the few study to evaluate the feasibility and safety of using TIVAP for blood collection in pediatric patients with ALL, which demonstrated important clinical benefits. It helps reduce physical and mental stress caused by repeated needle sticks in children. This directly improves their comfort during treatment and supports a better quality of life, which aligns with the National Comprehensive Cancer Network guidelines emphasizing minimally invasive monitoring strategies for pediatric hematologic malignancies. Fewer needle sticks mean lower risks of accidental exposure to bloodborne pathogens, protecting medical staff safety. Additionally, it saves time, eases workload and relieve job-related stress for nurses.

### Strength and Limitations:

In the present study, we assessed the feasibility of collecting blood samples using a TIVAP device that was concurrently used for methotrexate and electrolyte administration, which eliminated the need for additional venipuncture and significantly reduced the physical burden on pediatric patients. Compared with traditional peripheral venous blood sampling, no statistical differences were observed in laboratory parameters, and no adverse events were reported, indicating the feasibility and safety of this approach. In addition, the self-contemporaneous control design strengthens the validity of our findings by eliminating confounding factors inherent in cross-sectional comparisons. By using each patient as their own control, we ensured that observed differences were attributable to the sampling method rather than biological heterogeneity.

### Limitations

However, some limitations should be acknowledged. Although the sample size of 50 participants represents a relatively large cohort for studies of this type and provides adequate statistical power, the subgroup analyses based on disease severity (low, intermediate, and high risk) maybe constrained by relatively small sample sizes, potentially limiting the generalizability of our findings. In addition, although the observed coagulation abnormalities in the control group suggest a potential advantage of TIVAP, the small sample size of these events (n=2) limits its generalizability. Our cohort size may have constrained the detection of rare complications. Future multicenter studies with larger cohorts are warranted to validate these preliminary observations and establish risk stratification protocols.

## CONCLUSIONS

This comparative study demonstrated that TIVAP-based blood sampling exhibited equivalent clinical feasibility and safety profile compared to conventional peripheral venipuncture in pediatric ALL patients receiving HD-MTX therapy. We therefore recommended TIVAP as a preferred blood collection approach for children undergoing HD-MTX-based ALL protocols, particularly benefiting those with poor venous access or requiring long-term maintenance therapy.

### Author’s Contribution:

**YW and GB:** Study conceptualization and design.

**YW:** Collected data.

**MG:** Carried out statistical analyses and prepared tables/figures.

**YW and GB:** Prepared the main manuscript.

**GB:** Supervised the whole project and is also accountable for the accuracy or integrity of the work.

All authors have contributed to the interpretation of the data and approved the final version.

***Note:*** DeepSeek (an open source language model developed by Hangzhou DeepSeek Artificial Intelligence Co., Ltd.) was used for improvement of English language.
